# Health Care Professionals’ Knowledge, Attitude, Practice, and Infrastructure Accessibility for e-Learning in Ethiopia: Cross-Sectional Study

**DOI:** 10.2196/65598

**Published:** 2025-09-25

**Authors:** Sophie Sarah Rossner, Muluken Gizaw, Sefonias Getachew, Eyerusalem Getachew, Alemnew Destaw, Sarah Negash, Lena Bauer, Eva Susanne Marion Hermann, Abel Shita, Susanne Unverzagt, Pablo Sandro Carvalho Santos, Eva Johanna Kantelhardt, Eric Sven Kroeber

**Affiliations:** 1 Global and Planetary Health Working Group, Institute of Medical Epidemiology, Biometrics, and Informatics, Center of Health Sciences, Medical Faculty of the Martin Luther University Halle-Wittenberg, Magdeburger Str. 8, Halle (Saale), 06112, Germany; 2 Department of Epidemiology and Biostatistics, School of Public Health, Addis Ababa University, Addis Ababa, Ethiopia; 3 NCD working group, School of Public Health, Addis Ababa University, Addis Ababa, Ethiopia; 4 Institute of General Practice and Family Medicine, Center for Health Sciences, Martin-Luther-University Halle-Wittenberg, Halle (Saale), Germany; 5 Department of Gynecology, Martin-Luther-University Halle-Wittenberg, Halle (Saale), Germany

**Keywords:** e-learning, electronic learning, health care professional, education, medical education, medical learning, physician, doctor, primary care, primary health care, digital, digital health, digital technology, digital intervention, Ethiopia, Ethiopian, cross-sectional study

## Abstract

**Background:**

Training of health care professionals and their participation in continuous medical education are crucial to ensure quality health care. Low-resource countries in Sub-Saharan Africa struggle with health care disparities between urban and rural areas concerning access to educational resources. While e-learning can facilitate a wide distribution of educational content, it depends on learners’ engagement and infrastructure.

**Objective:**

This study aims to assess knowledge, attitude, practice, and access to infrastructure related to e-learning among health care professionals in primary health care settings in Ethiopia.

**Methods:**

In April 2023, we carried out a quantitative, questionnaire-based cross-sectional study guided by the knowledge, attitudes, and practice framework, including additional items on available infrastructure. The scores in each category are defined as “high” and “low” based on the median, followed by the application of logistic regression on selected sociodemographic factors. We included health care professionals working in general and primary hospitals, health centers, and health posts.

**Results:**

Of 398 participants (response rate 94.5%), more than half (n=207, 52%) reported feeling confident about their understanding of e-learning and conducting online searches, both for general (n=247, 62.1%) and medical-related content (n=251, 63.1%). Higher levels of education were associated with better knowledge (adjusted odds ratio [AOR] 2.32, 95% CI 1.45-3.68). Regardless of financial and personal efforts, we observed a generally positive attitude. Almost half of the participants (n=172, 43.2%) reported using the internet daily, compared to 16.8% (n=67) of participants who never used the internet. Higher education (AOR 2.56, 95% CI 1.57-4.16) and income levels (AOR 1.31, 95% CI 1.06-1.62) were associated with higher practice scores of e-learning-related activities. Women, however, exhibited lower practice scores (AOR 0.44, 95% CI 0.27-0.71). Regular access to an internet-enabled device was reported by 43.5% (n=173) of the participants. Smartphones were the primarily used device (268/393, 67.3%). Common barriers to internet access were limited internet availability (142/437, 32.5%) and costs (n=190, 43.5%). Higher education (AOR 1.56, 95% CI 0.98, 2.46) and income (AOR 1.50; 95% CI 1.21-1.85) were associated with increased access to infrastructure, while it was decreased for women (AOR 0.48, 95% CI 0.30-0.77).

**Conclusions:**

Although Ethiopian health care professionals report mixed levels of knowledge, they have a positive attitude toward e-learning in medical education. While internet use is common, especially via smartphone, the access to devices and reliable internet is limited. To improve accessibility, investments in the digital infrastructure and individual digital education programs are necessary, especially targeting women and those with lower income. Due to their widespread availability, e-learning programs should be optimized for smartphones.

## Introduction

### Background

#### Importance of Continuous Professional Development

Medical education is a fundamental part of a country’s health care system [1]. Continuous professional development (CPD) and continuous medical education are used to regularly update and enhance health care professionals’ knowledge and skills [2,3]. While CPD programs are seen as beneficial in low-and middle-income countries (LMICs) [4], their availability is often limited due to financial and personnel resources. This shortage of training opportunities for health care professionals affects the quality of medical care and sets patients’ health at risk [5].

#### e-Learning as a Globally Endorsed Strategy

The World Health Organization (WHO) endorses e-learning as a key component of their learning strategy, with the goal of reaching 3 million health care professionals by 2028 through approximately 260 courses. This offer leverages advanced multilingual technology and strategic partnerships to ensure accessible, culturally relevant, and impactful health education worldwide [6]. The WHO has established the “WHO Virtual Campus,” which offers tailored training programs, emphasizing the importance of conducting thorough needs assessments involving local health authorities and stakeholders prior to implementing e-learning initiatives [7]. The United Nations Online Learning Framework emphasizes the importance of understanding learners’ background, experiences, motivations, and infrastructural conditions before implementing online learning [8].

#### Advantages of e-Learning

The advantages of e-learning include improving the access to education [9], particularly in geographically or economically isolated areas [10], and being cost-effective, easy to update while improving the user’s performance and knowledge [11,12]. In addition, as a CPD strategy, e-learning has proven effective in enhancing both knowledge and procedural skills. Studies indicate that medical, surgical, and pharmaceutical (partly blended) e-learning courses received high levels of satisfaction and were considered useful by participants, even in low-resource settings [13-15]. e-Learning approaches have advanced health care quality and accessibility on a global scale [16].

#### Barriers in LMICs and Africa

Nevertheless, e-learning approaches are not universally suitable for all learners due to the diverse and individual nature of learning styles [17]. Their implementation often faces challenges in many LMICs with unique aspects in Africa compared to other parts of the world [18]. One of the main hurdles to implementing e-Learning projects on the continent is the low internet coverage of 43.2%, compared to 66% worldwide (2021) [18-20]. In Ethiopia, the internet penetration rate has slightly increased in recent years, reaching 19.4% in 2024 [6]. There is also a strong disparity in access to grid electricity between rural and urban areas. While almost all urban residents (95.7%) have access, only 43.6% of residents in rural areas are covered (2023 [21]). The lack of technical devices further disadvantages rural populations compared to urban residents [22], and as educational strategies shift toward distance learning, individuals in rural areas face even greater challenges in accessing education [23,24]. Further common barriers are costs, a lack of digital skills and knowledge, and computer fear [11,25-28]. The literature notes that Ethiopian educational institutions and students are insufficiently prepared for e-learning and have only used these platforms to a limited extent. This is mainly due to their focus on traditional education systems, which has resulted in a lack of efficient e-learning approaches. As a consequence, the development and adoption of modern digital learning methods remain restricted [21,22]. A systematic review (published in 2023) of challenges associated with e-learning in Sub-Saharan Africa noted that existing problems remained similar since 2016 without notable structural improvements [29].

#### Implementation Efforts in Africa

Several studies report on piloting and partly adopting e-learning in medical education. In Uganda, despite relevant efforts, nationwide implementation remains limited, with progress largely dependent on the commitment of individual institutions and educators, leading to uneven advancement across the country [30]. Many e-learning pilot projects are insufficiently adapted to local environments in LMICs [1], for example, addressing unreliable access and limited technological infrastructure [31]. This includes the usage of appropriate digital platforms and providing user support for a successful learning experience. Despite this, recruiting skilled personnel capable of managing and using e-learning effectively requires additional financial investment [9]. A baseline and needs assessment of the target group and area, as well as pretests, can identify strengths and limitations of e-learning tools and enable their adjustment to specific settings. Since e-learning is anticipated to be used increasingly in medical education worldwide, efforts are needed to be worthwhile to understand the target groups [32].

#### Rationale

The knowledge, attitudes, and practices (KAP) framework is a widely used approach to assess target groups in the context of public health [33]. It can be used to identify educational gaps and inform the design of targeted interventions, such as e-learning strategies. KAP studies provide a valuable foundation for tailoring digital learning tools to the specific needs, perceptions, and practices of diverse user groups. In our literature review, we did not find publications from LMICs or Sub-Saharan Africa specifically concerning the perspective of health care professionals regarding e-learning, highlighting the need to address this gap [34]. The existing literature mainly focuses on students enrolled in universities and colleges during the COVID-19 pandemic [28,35,36], as well as individuals among the general population [37].

### Objective

Our study aims to assess knowledge, attitude, practice, and access to infrastructure (KAP-Infrastructure) related to e-learning courses among health care professionals working at the primary health care level in Central and Southern Ethiopia. Our study provides an opportunity to offer insights and address the existing gaps and missing information in this area and setting.

## Methods

### Overview

We conducted a data collector–supported self-administered cross-sectional survey among health care professionals working as general practitioners, health officers, nurses, midwives, or health extension workers in 45 health facilities between April 3 and 28, 2023. The study was set in the Oromia region and the former Southern Nations, Nationalities, and Peoples Region (SNNPR), which was reorganized in August 2023 after the data collection. The study regions are now part of the South Ethiopia Regional State and the Central Ethiopia Regional State. More than 85% of residents in Oromia and 89% of residents in the former SNNPR live in rural areas [38,39]. The selection of the health care facilities (2 general hospitals, 3 primary hospitals, 14 health centers, and 26 health posts) was based on partner settings in a collaborative cervical and breast cancer care project between the Addis Ababa University School of Public Health, Ethiopia and the Martin Luther University Halle-Wittenberg, Germany. Ethiopia has a 3-tier health care system with specific responsibilities on each level to enable continuous care in the country (Multimedia Appendix 1). At the primary health care level, health posts, health centers, and primary hospitals deliver essential health care services, particularly in rural areas [23], where 77.4% of the Ethiopian population is resided. General and referral hospitals, as part of the secondary health care level, provide a wider range of medical services and treatments. Specialized hospitals form the tertiary level and provide extensive services for comprehensive health care [40]. We included 2 general hospitals as part of the secondary health care level to perform a sensitivity analysis comparison between health care levels (see Multimedia Appendix 1). Our study area is located approximately 150 km to the south and southwest of the capital, Addis Ababa, in a radius of about 100 km.

### Outcome Variables

We investigated the 4 KAP-Infrastructure constructs, building items based on the following definitions (1) “Knowledge” assessed the health care professionals’ self-perceived practical abilities related to e-learning tasks. (2) “Attitude” represented participants’ motivation and expectations toward e-learning to support their professional education. (3) “Practice” concerned the conduct of activities related to e-learning. Finally, (4) “Access to infrastructure” covered individual and work-related infrastructural conditions of the health care professionals.

### Questionnaire

The questionnaire contained questions on sociodemographic characteristics as well as the participants’ perspectives on e-learning based on the KAP model [33]. The KAP items had 5-step Likert scale answering options [41].

Besides using the United Nations Online Learning Framework as an orientation, we covered relevant aspects of e-learning by using items described in a systematic review outlining enablers and barriers affecting e-learning in health sciences [11]. We added a category “access to infrastructure” (KAP-Infrastructure) as a relevant aspect for e-learning conduct. We based this section on the European Union’s “Community survey on ICT usage in households and by individuals (2020)” [42], with additional items adapted from a community survey on information and communication technology [35,37] and a KAP study on online learning by college students in India [35].

The questionnaire contained 10 sociodemographic, 10 knowledge, 13 attitude, 8 practice, and 7 infrastructure items. It was initially developed in English and then translated into the Amharic and Afan Oromo languages. A blind back-translation into English ensured accuracy. Discrepancies were addressed and discussed. The main translation process involved 5 native speakers of Amharic and Afan Oromo, all of whom were proficient in English and had a medical background.

Due to the lack of a topically fitting validated research instrument, the questionnaire was developed by an interprofessional team of health scientists from Martin Luther University Halle-Wittenberg and Addis Ababa University based on comprehensive initial literature research. We performed pretests according to the Concurrent Think Aloud Method and adapted the questionnaire accordingly, ensuring its comprehensibility, suitability, and validity. Two rounds of pretests were conducted in Addis Ababa in March 2023, including 38 health care professionals in 2 health centers. These participants represented approximately 9% of the overall sample, closely meeting the targeted 5% per health care center. The items were tested for understandability and clarity in the Amharic and Afan Oromo languages. The received feedback was then reviewed and discussed by interdisciplinary teams in both Germany and Ethiopia, resulting in the exclusion or rephrasing of certain questions and answer choices, as well as modifications to the consent section. The process was guided by “Surveys and Questionnaires in Health Research” by Schofield and colleagues [43,44].

### Sample Size and Sampling Technique

Before collecting the data, the sample size was calculated using the single population proportion formula. The level of KAP of health care professionals on e-learning in Ethiopia has not been studied yet. Therefore, the sample size was calculated with the some assumptions, including 50% proportion of the outcome among health care professionals, 5% marginal error, and 95% CI. Under these circumstances, the sample size is 384, and after adding a 10 % nonresponse rate, the final sample size was 423. For comparison, we also calculated the sample size to estimate a 2-sided CI for one proportion for the target variable “internet access” as a representation for the objective “infrastructure.” With this frame we calculated a sample size of 395, including a 10% nonresponse rate. Since the calculated sample size of infrastructure was smaller than the sample size of KAP, we decided to achieve a larger sample size of 423. We selected participating health care professionals working in the affiliated health facilities using the convenience sample method and reached a final sample size of 398 participants (response rate 94.1%). All health care professionals, including general practitioners, health officers, nurses, midwives, and health extension workers present during data collection, were included in the study. However, those who were unavailable at that time and any professionals not listed above were excluded from the study. Ensuring a balanced participation between all occupations, we considered a proportional allocation for each health facility.

### Data Analysis

First, we conducted basic descriptive statistics. After building sum scores for each KAP-Infrastructure category, the median was used to define “low” and “high” scores in each category, which were considered as binary response variables in downstream analyses (Multimedia Appendix 2). In order to detect potential monotonic relationships (collinearity) among predictor variables, we used Spearman rank correlation coefficient (ρ) [45] (refer Multimedia Appendix 3). This preliminary diagnostic analysis yielded a high correlation between the variables “health facility” and “occupation” (ρ=0.982). As a ρ above 0.9 is generally considered very high, we proceeded to remove the variable “occupation” from all multivariate logistic regressions in order to avoid redundancy and ensure convergence of statistical modeling. The remaining 7 explanatory variables were kept in all multivariate logistic regressions. We divided the participants into 5 similar-sized income groups (monthly income less than US $94, US $94‐US $115, US $115‐US $130, US $130‐US $151.2, and more than US $151.2 USD), according to the World Bank’s country income classifications [46]. We applied a more precise breakdown that is often used in household or individual-level analyses by adding middle income. The income thresholds were established to produce comparable income ranges across different income-level groups. We defined regular usage of the internet and internet access as an available internet service in terms of messaging, browsing, downloading, and purchasing in the last 3 months for at least once a week [47-49]. Data imputation was carried out based on Spearman correlation indices among the variables. It was done for 0.7% of data points in the “religion” and “sex” variables and for 3.8% of data points in the “income” variable. The correlation indices among the components of the dependent variable (KAP-Infrastructure scores) were used to allow imputation as well, which led to 1.1% of data points in the dependent variables being imputed. We then investigated the relationship between KAP-Infrastructure score and multiple explanatory factors (age, sex, education level, income, and type of health facility) by calculating odds ratios (ORs) and adjusted ORs (AORs) and the corresponding 95% CI with a logistic regression, using SPSS version 28 (IBM Corp) [50]. An OR greater than 1 indicates that the factor is associated with higher KAP-Infrastructure scores. We tested each category for internal consistency by calculating Cronbach α (knowledge 0.897; attitude: 0.688; practice: 0.899; access to infrastructure: 0.810) [51]. The results indicated acceptable or high internal consistency. Furthermore, we assessed the fit of a logistic regression model using the Hosmer-Lemeshow test [52].

### Ethical Considerations

We received approval from the Addis Ababa University Research Ethics Committee in Addis Ababa, Ethiopia (Prev: 423), and the ethics committee of the Martin Luther University Halle-Wittenberg-Medical Faculty (processing number 2023‐003). Participant information was collected anonymously, and written consent was obtained in the questionnaire. Participants did not receive any compensation.

## Results

### Overview

We included a total of 398 participants (response rate, 94.1%). Overall, 25 health care professionals declined due to workload, unwillingness, or undisclosed reasons. Male (195/394, 49.5%) and female (199/394, 50.5%) participants were equally represented. The mean age was 31.0 (SD 7.0) years. Half of the health care professionals were nurses (200/398, 50.3%). In total, 86.7% (345/398) of the participants chose the Amharic version of the questionnaire. The mean income per month was US $138 (SD $57). Among the participants, 4 out of 5 worked in primary facilities (316/398, 79.4%; Table 1).

**Table 1.** Sociodemographic information.

Characteristic and category		Value	
Region (N=398), n (%)			
SNNPR^a^		252 (63.3)	
Oromia region		146 (36.7)	
Type of health facility (N=398), n (%)			
Health post		31 (7.8)	
Health center		158 (39.7)	
Primary hospital		127 (31.9)	
General hospital		82 (20.6)	
Occupation (N=398), n (%)			
Nurse		200 (50.3)	
Midwife		68 (17.1)	
Health extension worker		30 (7.5)	
Health officer		61 (15.3)	
General practitioner		39 (9.8)	
Age in years (N=390) mean (SD); range		31.0 (7.0); 20.0-64.0	
Sex (N=394), n (%)			
Female		199 (50.5)	
Male		195 (49.5)	
Education level (N=396), n (%)			
Diploma		151 (38.1)	
Bachelor of Science		208 (52.5)	
Master of Science and above		37 (9.3)	
Income group in US ($; per month; N=383)^b^, n (%)			
<94		77 (19.3)	
94-115		77 (19.3)	
115-130		73 (18.3)	
130-151.2		70 (17.6)	
>151.2		86 (21.6)	
Chosen language of the questionnaire (N=398), n (%)			
Amharic		345 (86.7)	
Afan Oromo		53 (13.3)	

a Former Southern Nations Nationalities and Peoples Region.

b Exchange rate of 1 Ethiopian Birr to US $0.0167, as of April 16, 2023.

### Knowledge

**Figure 1.**
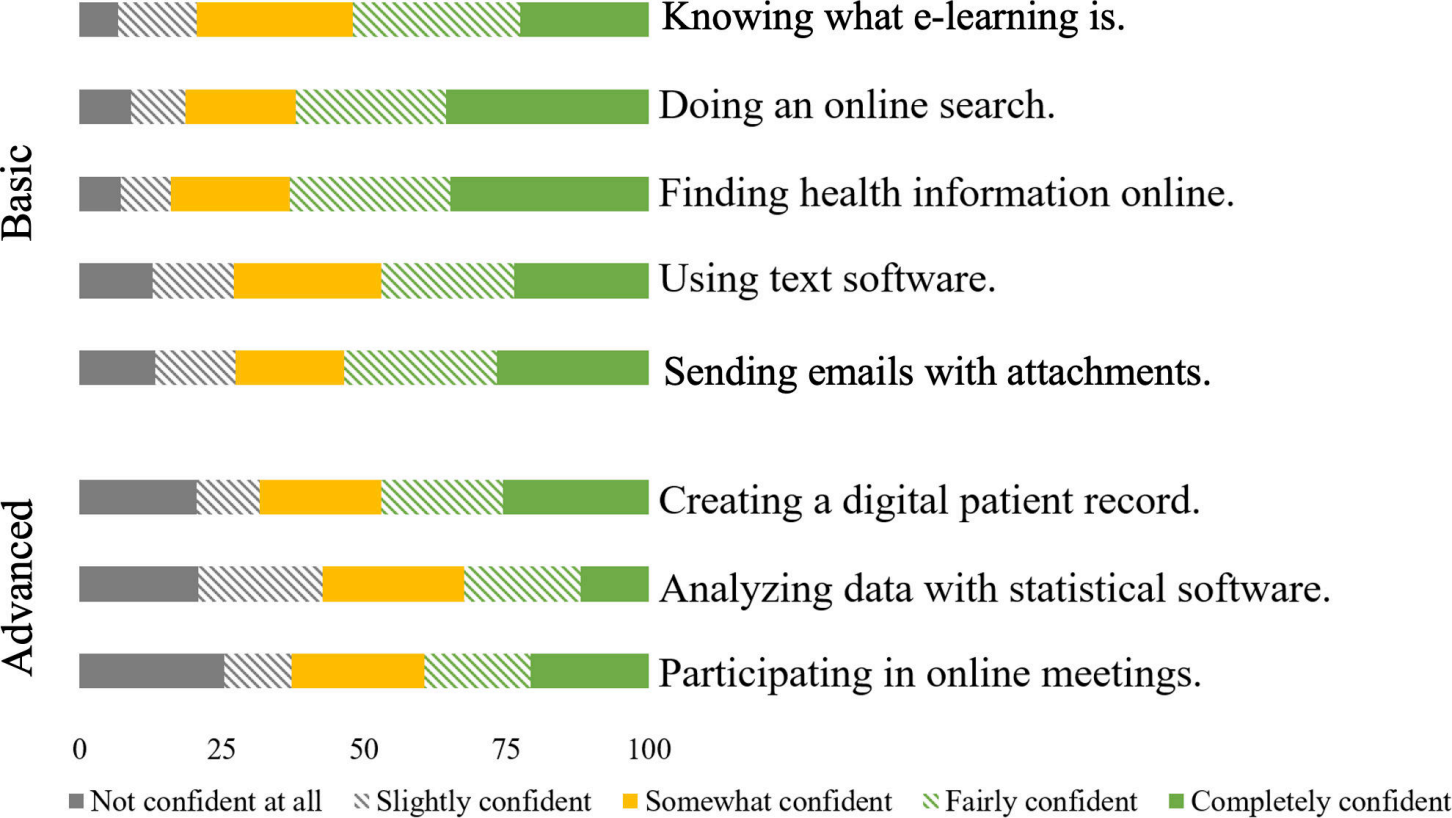
Self-perceived knowledge (%).

About half of the health care professionals were completely (90/398, 22.6%) or fairly confident (117/398, 29.4%) in knowing what e-learning is. Almost two thirds of them described a self-perceived competence in doing general (247/398, 62.1%) and medical-related (251/398, 63.1%) online searches. Over one-third were not or slightly confident in their ability to participate in online meetings (157/398, 37.2%; Figure 1).

### Attitude

**Figure 2.**
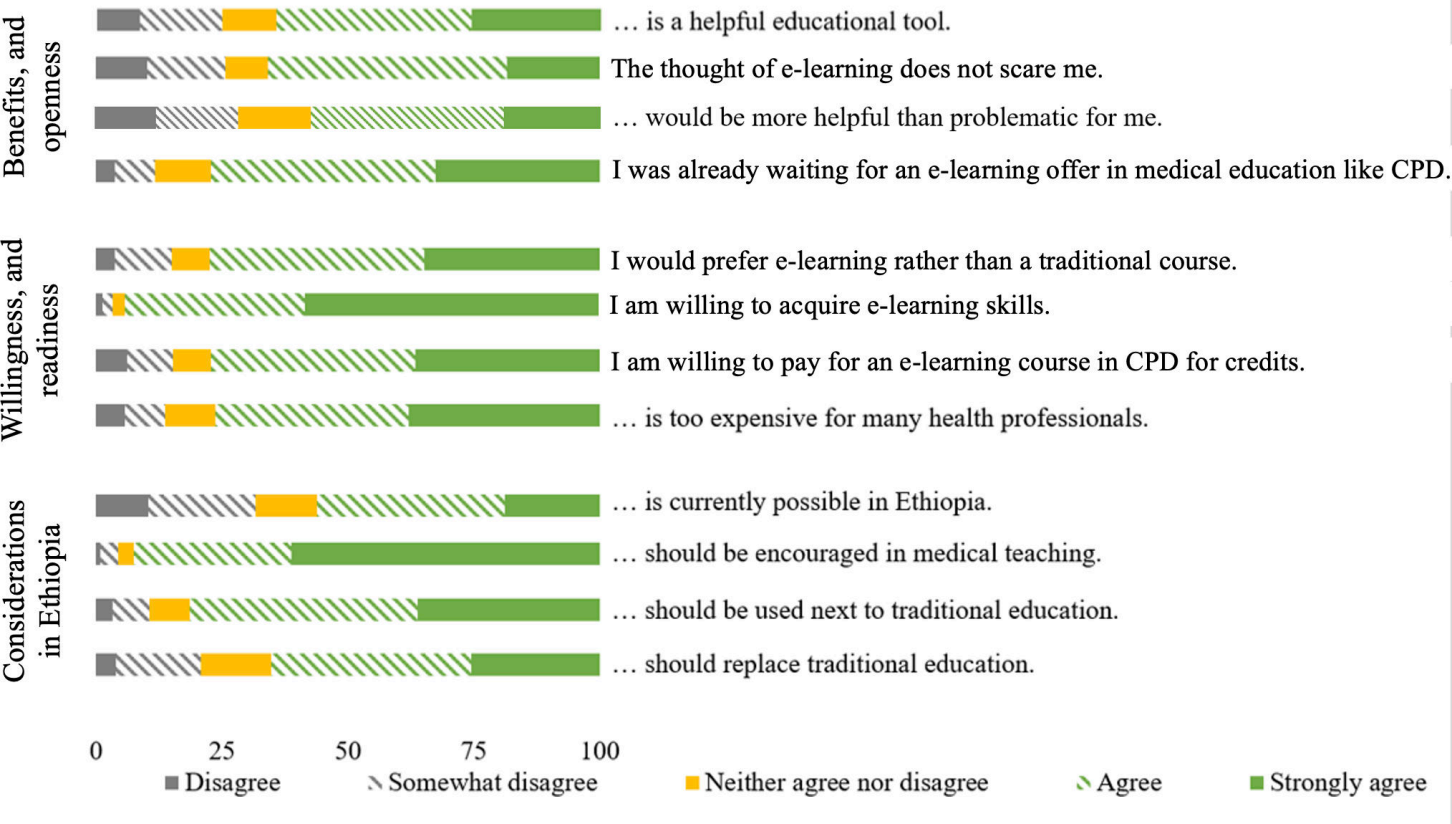
Health care professionals’ attitude toward e-learning (%). CPD: continuous professional development.

Health care professionals predominantly had a positive attitude toward e-learning, with 94.2% (375/398) willing to put effort into acquiring e-learning skills. More than half (224/398, 56.2%) believed that e-learning is possible in rural parts of Ethiopia. Almost all health care professionals were in favor of integrating e-learning in medical teaching institutions (368/398, 92.5%). Some participants expressed fear (102/398, 25.7%) or perceived e-learning as more problematic than helpful (113/398; 28.4%; Figure 2).

### Practice

**Figure 3.**
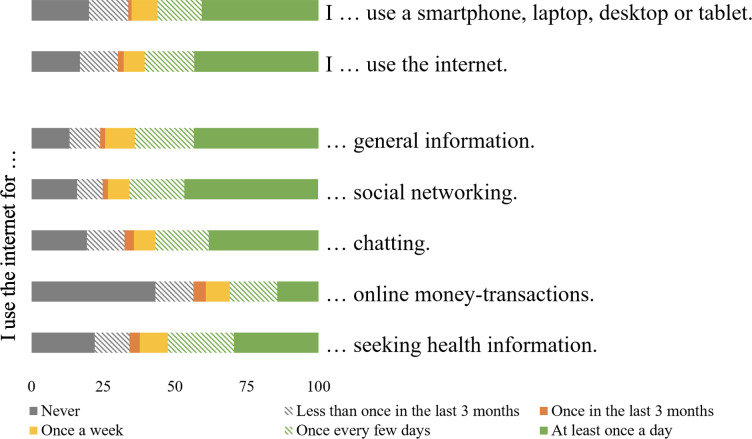
Practice of e-learning (%).

Daily internet usage was reported by 172/398 (43.2%) participants and 67/398 (16.8%) participants never used it. More than half used the internet for general (255/398, 64.1%) and medical-related searches (209/398, 52.5%), social networking (262/398, 65.8%), and chatting (226/398, 56.8%). In total, 2 out of 3 health care professionals stated a regular usage (at least once a week) of an internet device (259/398, 65.1%) and pointed out smartphones as the most used device (268/393, 67.3%; Multimedia Appendix 4 and Figure 3).

### Access to Infrastructure

**Figure 4.**
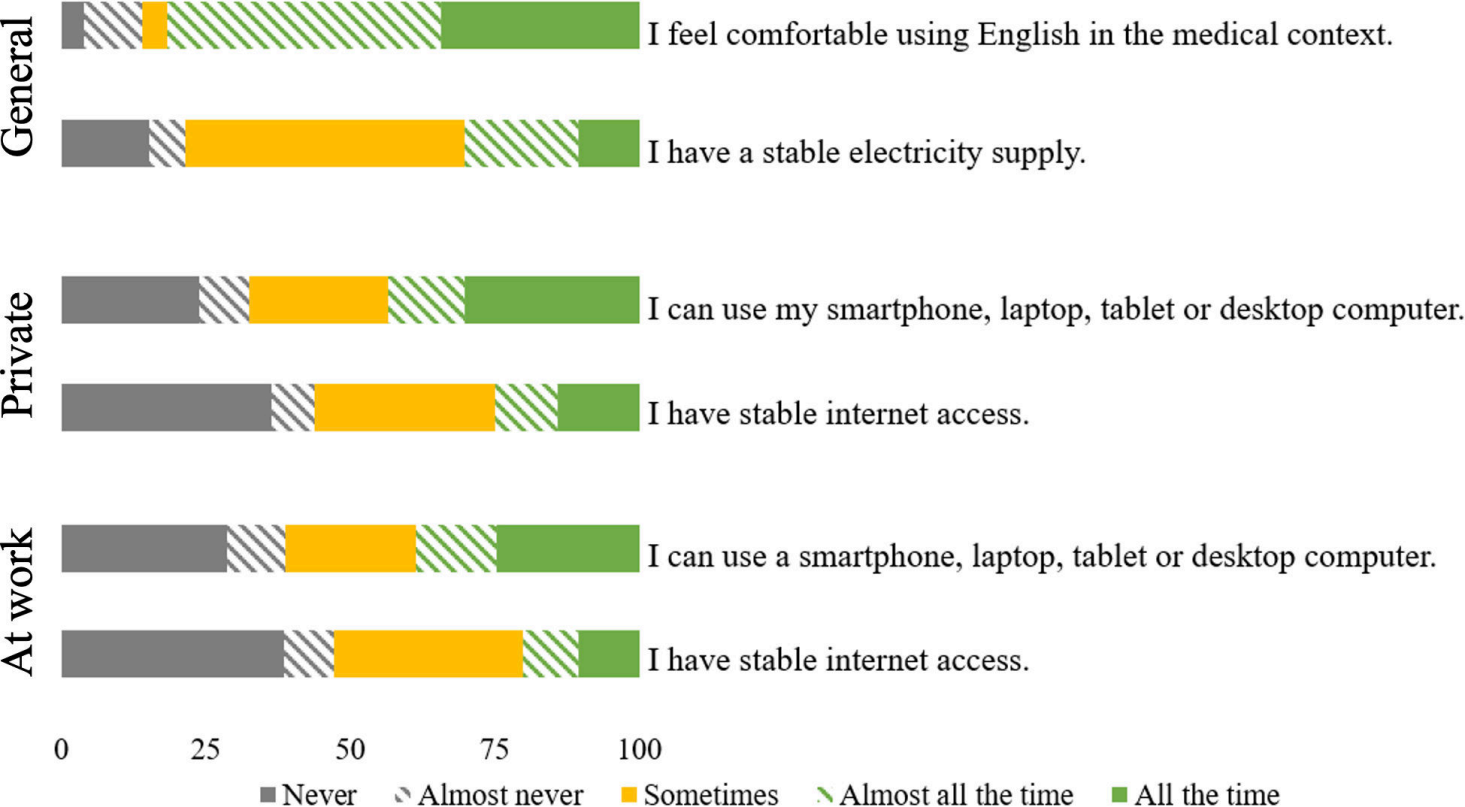
Health care professionals’ access to infrastructure (in %).

Access to digital devices (154/398, 38.7%) and stable internet (80/398, 20.1%) at the participants’ workplace was less common than at home (access to private devices [173/398, 43.5%] and stable internet [99/398, 24.9%]). Common barriers included problems with internet availability (142/437, 32.5%) and internet costs (190/437; 43.5%; Multimedia Appendix 5 and Figure 4).

### Factors Associated With Knowledge, Attitude, Practice, and Infrastructure

Higher levels of education were associated with increased knowledge (AOR 2.31, 95% CI 1.45-3.68), whereas higher age was associated with lower knowledge (AOR 0.94, 95% CI 0.91-0.97). Health care professionals with a higher education level (AOR 2.56, 95% CI 1.57-4.16) and income group (AOR 1.31, 95% CI 1.06-1.62) were more likely to have higher practice scores. Female (AOR 0.44, 95% CI 0.6-0.71) and older participants (AOR 0.91, 95 % CI 0.87-0.95) were likely to report lower practice scores. Participants from higher income groups (AOR 1.50, 95% CI 1.21-1.85) had increased odds of having higher access to infrastructure. For detailed information see Table 2 and MultimediaAppendices 6  7.

**Table 2.** Factors associated with knowledge, attitude, practice–infrastructure.

Variable	Self-perceived knowledge, AOR^a^ (95% CI)	Attitude, AOR (95% CI)	Practice, AOR (95% CI)	Access to infrastructure, AOR (95% CI)
Age (years)	0.94 (0.90-0.97)	1.01 (0.98-1.04)	0.91 (0.87-0.95)	0.94 (0.91-0.98)
Female sex^b^	0.69 (0.43-1.11)	1.01 (0.65-1.56)	0.44 (0.67-0.71)	0.48 (0.30-0.77)
Higher education level^c^	2.31 (1.45-3.68)	1.19 (0.78-1.82)	2.56 (1.57-4.16)	1.56 (0.98-2.46)
Higher income group	1.16 (0.95-1.41)	1.06 (0.88-1.28)	1.31 (1.06-1.62)	1.50 (1.21-1.85)
Health facility^d^				
Health post	2.00 (0.86-4.66)	1.48 (0.65-3.38)	0.70 (0.25-2.00)	0.31 (0.11-0.90)
Primary hospital	0.66 (0.39-1.09)	1.12 (0.69-1.78)	0.61 (0.36-1.04)	0.55 (0.33-0.91)
General hospital	1.97 (1.08-3.60)	0.60 (0.34-1.03)	1.01 (0.54-1.86)	1.08 (0.59-1.98)

a AOR: adjusted odds ratio.

b Male as reference.

c Diploma<Bachelor of Science<Mater of Science.

d Health center as reference.

## Discussion

### Principal Findings

The majority of the participants knew about e-learning and viewed themselves as competent in different e-learning-related activities. Still, we found difficulties in relevant knowledge aspects like participating in online meetings or searching for medical information. Moreover, a high proportion of workers had a positive attitude toward the use of e-learning. Almost half of the participants stated using the internet daily, whereas 16.8% (67/398) never use the internet. Infrastructure, such as technical devices and stable internet, was more often available at home than at work. The availability of stable internet was reported at below 25% (at home: 24.9% and at work: 20.1%).

Health care professionals with higher levels of education and income and those working in general hospitals had better KAP of e-learning and better access to infrastructure. Older health care professionals, women, and those working in health posts and primary hospitals reported reduced access to infrastructure.

### Strengths and Limitations

First, the included health facilities show differences in terms of health service quality, with some having been rewarded international awards and not necessarily representing the typical peripheral hospital in Ethiopia. However, this diversity is part of the primary health care setting and should be represented in the study.

Second, during the data collection, we observed that some participants were confused about the definition of a smartphone, occasionally mistaking it for a tablet. These cases were clarified by the data collectors. Despite verification and testing of the translated questionnaire, we cannot completely rule out cases of misinterpretation. However, it is not expected to significantly affect the results.

Third, the health care facilities are exclusively located in Oromia and the former SNNPR region. Therefore, the results may not be generalizable for Ethiopia. While future research should investigate regional differences, it is reasonable to assume that our data from the context of primary health care settings are relevant and transferable to other parts of the country to a certain extent. Given the absence of studies in comparable settings, our data offer valuable insights for the development and implementation of effective e-learning courses.

### Comparison With Prior Work

Participants report mixed levels of confidence regarding knowledge or e-learning–related tasks. More than 3 quarters of the health care professionals are at least somewhat confident in understanding the concept of e-learning (316/398, 79.4%), in performing basic digital tasks, such as general online searches (324/398, 81.4%), in finding professional health information on the internet (334/398, 83.9%), or in using writing programs, such as Microsoft Word (290/398, 72.9%). We did not find literature specifically targeting the knowledge of e-learning of health care professionals in LMICs, but the Ethiopian health care professionals were less confident in basic tasks, such as sending email attachments (53.5%) and conducting online searches (62.1%) compared to medical undergraduate students in India. Both studies identified creating patient records as particularly challenging [36]. College students in India and Pakistan reported experiencing technical difficulties due to a lack of knowledge on e-learning. Nevertheless, they had an overall acceptable level of knowledge regarding media content and communication [28,35,53]. Possible reasons could include the higher internet penetration rate in India (52.4%) compared to Ethiopia (26.7%; 2024) [54,55]. This could be one of the reasons consequently leading to this difference.

Moreover, the lack of digital knowledge remains a significant challenge, further widening the digital divide between rural and urban areas [56,57]. Addressing these barriers is essential. Therefore, offering basic digital training programs can play a crucial role in bridging this gap [56]. Older health care professionals, those with lower education, and participants working outside general hospitals should be supported when implementing e-learning programs. A study from Kenya offers a further perspective on e-learning knowledge. Medical trainees faced difficulties in completing the courses due to limited technical skills and a lack of engagement from trainers and institutions responsible for developing the content. In addition to the extensive volume of e-learning materials and frequent online meetings, the courses contributed to feelings of being overwhelmed among the students. Approximately half of the students included in the study did not finish the course. Consideration should be given not only to the participants’ knowledge but also to the content creators’ [58].

Despite these mixed results, the overall attitude among Ethiopian health care professionals remains positive, and many even consider making financial and personal investments to support its implementation. In contrast, students from Uganda and Turkey showed an overall negative attitude toward e-learning. Participants’ income, internet quality, ownership of technical devices, and previous use of academic sites were associated with attitude scores [59,60]. In Pakistan, similar issues did show, including technical and infrastructural challenges, limited technical skills, and the general disadvantages of e-learning, such as decreased connection and interaction with other participants, led to widespread dissatisfaction among the surveyed students. Consequently, 50% of them frequently missed e-learning classes. Nearly 80% considered e-learning less effective than traditional classroom instruction, particularly when it comes to acquiring practical skills [61]. Health care professionals in urban Taiwanese settings had a less positive attitude compared to those in rural settings but still valued the increase in knowledge, time saving, and diverse, and wide offer of information [62]. Medical students in Sudan favored a combination of electronic and traditional teaching methods [63]. Similarly, most of our participants (81.4%) are in favor of e-learning as an addition to traditional courses, even expressing a preference of e-learning over traditional workshops and lectures (77.4%). Overall, our results indicate that Ethiopian health care professionals are motivated to use e-learning for their professional development.

These positive attitudes can support the success of educative efforts [64], including e-learning [65]. e-Learning requires a higher level of self-motivation compared to other educational concepts [66], especially for participants with limited experience [67]. Incorporating feedback and evaluation is crucial in addressing these issues [11]. In addition, the rapidly evolving technical standards necessitate a positive attitude to continuously improve one’s skills [68]. However, since a relevant share of health care professionals expressed fear, preparatory courses are advisable [1].

Regarding the practice of e-learning-related activities, almost half of the health care professionals (172/398, 43.2%) reported using the internet daily, which is less frequent than nursing students (68.1%) and comparable to patients from Gondar, Ethiopia (47%) [69,70]. Similar to our findings, being young, male, more educated, and with an urban residence is associated with increased internet use [70]. In rural communities in Sub-Saharan Africa, the internet is primarily used for browsing (13.9%) and email communication (13.2%), followed by information search (12.5%), chatting (10%), social networking (9.6%), and video conferencing (5.8%) [71]. Undergraduate students from Ethiopia with a rural background predominantly used the internet for social media [69]. Similarly, our participants most commonly used the internet for social networking and chatting, but also for general and medical searches. While a majority of primary health care professionals reported frequent internet use and engagement in e-learning–related digital activities, there is a relevant share of health care professionals (39.5%) who reported less frequent internet use, hinting at potential difficulties in handling online learning formats. To address this, targeted support for lower-income groups, women, and less-educated health care professionals is recommended.

Notably, smartphones emerged as the most commonly used device for accessing infrastructure, which aligns with findings from other Ethiopian settings [70]. Mobile phone ownership and usage has increased in countries of the global south, including rural communities in Sub-Saharan Africa, although access is not ubiquitous [70,72]. Mobile phones were among the least commonly used devices for use in our study, ranking lower than smartphones, tablets, laptops, and other devices. Smartphones are becoming increasingly important and prevalent as part of mobile health (mHealth) initiatives [73]. The WHO [25], along with other authors [19,26,74] recommends smartphones as a possible platform for e-learning to reach remote parts of countries with low resources. Health extension workers and midwives working in primary health care facilities in Ethiopia perceived the use of mobile devices in an mHealth program as easy and beneficial. The program was implemented for both the health care providers themselves as well as for the patients [75]. The Ethiopian Ministry of Health also introduced a community-based health extension program, which incorporates mHealth [76]. Nevertheless, rural communities in Sub-Saharan Africa used the mobile phone majorly for receiving (89.8%) and making calls (88.5%) and the least for internet browsing (27.3%) [71]. A study conducted in 4 primary hospitals in Ethiopia in 2018 revealed that 12.1% of health care professionals had internet access and 25.6% had access to a computer at work. Private computers were more often accessible at 33.3% [53]. Our study similarly showed higher accessibility to private internet and technical devices compared to work. Lacking bandwidth and stable internet access are common limitations, resulting in slow speed and low quality of e-learning programs that may create difficulties for users to load digital content [9]. Power issues, shortage of skilled personnel for support, the cost of internet and necessary devices, and problems of viruses and malware can additionally hinder access to online education [77]. In addition, South Africa reports socioeconomic challenges that widen the digital divide and are thereby hindering the implementation of e-learning across various settings of the country [78]. For these reasons studies suggest e-learning courses with downloadable offline versions may help to establish accessible e-learning programs [79]. This increased accessibility and attempt to include students from different socioeconomic backgrounds, and in turn, positively influenced students’ acceptance of e-learning in Amhara, Ethiopia, by enhancing their perceived ease of use [80].

Regarding possible gender differences, female health carecprofessionals had lower scores in knowledge, practice, and access to infrastructure while displaying a similar attitude. This is consistent with reports on the digital divide, both globally and in Ethiopia, which can in part be attributed to gender inequality [81]. For example, in Ethiopia, 14% of females used the internet, compared to 20% of males [82], and women faced lower computer literacy and access to infrastructure [19]. To reduce these disparities, supporting women, including the development of knowledge and skills, as well as addressing aspects of the costs of communication technology is necessary [83,84].

### Conclusion

Health care professionals report mixed levels of knowledge in e-learning-related task while generally having a positive attitude toward e-learning in medical education. More than half use the internet regularly, especially via smartphones, while the access to digital infrastructure and reliable internet remains limited. Efforts should be made to improve access to internet access and technological infrastructure, as well as the adaptation of e-learning courses to the local needs. This could include the provision of introductory trainings, particularly for women and health care professionals with lower income and educationl; provision of downloadable offline learning options; and the adaptability of the course to smartphone use.

## Supplementary material

10.2196/65598 Multimedia Appendix 1 Distribution of participants according to health care level, region, and type of health facility.

10.2196/65598 Multimedia Appendix 2 Cutoff points for categorization of high and low knowledge, attitude, practice, and infrastructure.

10.2196/65598 Multimedia Appendix 3 Correlation matrix.

10.2196/65598 Multimedia Appendix 4 Most often used device.

10.2196/65598 Multimedia Appendix 5 Reasons for restricted internet access.

10.2196/65598 Multimedia Appendix 6 Complete table factors associated to knowledge, attitude, practice, and access to infrastructure.

10.2196/65598 Multimedia Appendix 7 Distribution of the answered questionnaire.

## Abbreviations

AOR: adjusted odds ratio
CPD: continuous professional development
KAP: knowledge, attitude, and practice
KAP-I: knowledge, attitude, practice, and infrastructure
LMIC: low- and middle-income country
mHealth: mobile health
OR: odds ratio
SNNPR: Southern Nations Nationalities and Peoples Region
WHO: World Health Organization
