# Supplementary material for: Health Care Professionals’ Knowledge, Attitude, Practice, and Infrastructure Accessibility for e-Learning in Ethiopia: Cross-Sectional Study
Source: JMIR Med Educ. 2025 Sep 25;11:e65598. doi: 10.2196/65598 (PMC12463343; doi:10.2196/65598)
Supplement: Multimedia Appendix 1 [file mededu-v11-e65598-s001.pdf]

	Primary Health Care Level				Secondary Health Care Level		Tertiary Health Care Level			
	Health Post (HP)	Health Center (HC)	Zone/Woreda	Primary Hospital	General Hospital	Referral Hospital	Specialized Hospital	Town of hospital	Region	Participants per region (n)
	Bati Futo HP	Enseno HC	Gurage/Meskan		Butajira General Hospital			Butajira	SNNPR	252
	Weja HP									
	Tawula HP	Worabe HC	Silte/Worabe				Worabe Comprehensive Specialized Hospital	Worabe		
	Beketa HP									
	Yeferzey HP	Hole HC	Gurage/Cheha	Attat Our Lady of Lourdes Catholic Hospital				Atat		
	Gerar HP									
	Dubancho HP	Shumo HC	Hadiya/Lemo				Nigist Eleni Mohammed Memorial Comprehensive Specialized Hospital	Hossana		
	Dejo HP									
	Kerekicho HP	Angacha HC	Kembata/Angacha					Angacha		

	1st Anigecha HP			Angacha Primary Hospital						
	Misrak Gortenicho HP	Arsho HC	Halaba/Wera		Halaba Kulito General Hospital			Halaba		
	Tache Arisho HP									
	Mante HP	Tome Gerara HC	Wolaita/Sodo Zuria			Wolaita Sodo University Teaching and Referral Hospital		Wolaita		
	Tome HP									
	Adamancho Arfita HP	Gara Godo HC	Wolaita/Boloso Sori	Dubo St. Mary Primary Hospital				Areka (no current data available)		
	Gara Godo HP									
	Aze Dolo HP	Bezena	Kembata/Kedida		Dr. Bogalech G. Memorial General			Durame		
	Jore HP									

					Hospital					
	Dalole HP	Korike	West Showa/Woliso		St. Luke Catholic Hospital			Woliso	Oromia	146
	Jelisa Cheka HP									
	Gadiya HP	Adulala HC	East Showa/Liben Chikuala			Adama Referral Hospital		Adama		
	Dodolo HP									
	Hunde Gudetu HP	Asela HC	Arsi/Assela			Asela Referral Hospital		Asela		
	Hnko HP									
	Kebele 04 HP	Cheleleka HC	East Showa/Bishoftu		Bishofitu General Hospital			Bishofitu		
	Kebele 05 HP									
	Heda HP	Guliso HC	West Wolega/Guliso		Aira General Hospital			Aira		
	Moga HP									
	Wolibilo HP	Dembel HC	East Shoa/Batu		Batu General Hospital			Batu		
	Edo Gejole HP									

Participants (n)	31	158		127	82					398
Participants (%)	7.8	39.7		31.9	20.6					
Included health facilities (n)	26	14		3	2					
