# Supplementary material for: Health Care Professionals’ Knowledge, Attitude, Practice, and Infrastructure Accessibility for e-Learning in Ethiopia: Cross-Sectional Study
Source: JMIR Med Educ. 2025 Sep 25;11:e65598. doi: 10.2196/65598 (PMC12463343; doi:10.2196/65598)
Supplement: Multimedia Appendix 2 [file mededu-v11-e65598-s002.pdf]

Category	Median as cut-off point (high / low)	Maximum score
**Knowledge**		
	24.0	40.0
**Attitude**		
	28.0	52.0
**Practice**		
	24.0	35.0
**Access to infrastructure**		
	12.0	24.0
