# Supplementary material for: Health Care Professionals’ Knowledge, Attitude, Practice, and Infrastructure Accessibility for e-Learning in Ethiopia: Cross-Sectional Study
Source: JMIR Med Educ. 2025 Sep 25;11:e65598. doi: 10.2196/65598 (PMC12463343; doi:10.2196/65598)
Supplement: Multimedia Appendix 5 [file mededu-v11-e65598-s005.pdf]

My internet access is restricted (at home or at work), because …		
	n	%
… the costs are too high.	190	43.5%
… internet is not available in my area.	142	32.5%
… of privacy and security concerns.	30	6.9%
None of the given answers. My internet access is not restricted.	30	6.9%
… I don´t have the required skills.	28	6.4%
… of other reasons.	17	3.9%
