# Supplementary material for: Health Care Professionals’ Knowledge, Attitude, Practice, and Infrastructure Accessibility for e-Learning in Ethiopia: Cross-Sectional Study
Source: JMIR Med Educ. 2025 Sep 25;11:e65598. doi: 10.2196/65598 (PMC12463343; doi:10.2196/65598)
Supplement: Multimedia Appendix 6 [file mededu-v11-e65598-s006.pdf]

Variable		**Self-perceived knowledge** of e-Learning		**Attitude** towards e-Learning		**Practice** of e-Learning		**Access to Infrastructure** for e-Learning	
		OR (95%-CI)		OR (95%-CI)		OR (95%-CI)		OR (95%-CI)	
		COR	AOR	COR	AOR	COR	AOR	COR	AOR
**Age in years**									
		**0.96 (0.93, 0.99)**	**0.94 (0.90, 0.97)**	1.02 (0.99, 1.05)	1.01 (0.98, 1.04)	**0.95 (0.92, 0.98)**	**0.91 (0.87, 0.95)**	0.99 (0.96, 1.01)	**0.94 (0.91, 0.98)**
**Sex**[i]									
	Female	**0.60 (0.41, 0.90)**	0.69 (0.43, 1.11)	0.94 (0.64, 1.39)	1.01 (0.65, 1.56)	**0.35 (0.23, 0.53)**	**0.44 (0.67, 0.71)**	**0.37 (0.24, 0.55)**	**0.48 (0.30, 0.77)**
**Education level**									
	Higher level (Diploma < Bachelor of Science < Mater of Science)	**2.70 (1.91, 3.82)**	**2.31 (1.45, 3.68)**	1.14 (0.83, 1.56)	1.19 (0.78, 1.82)	**3.94 (2.71, 5.75)**	**2.56 (1.57, 4.16)**	**3.19 (2.23, 4.57)**	1.56 (0.98, 2.46)

**Income group**									
	Higher income	**1.27 (1.10, 1.46)**	1.16 (0.95, 1.41)	1.09 (0.95, 1.25)	1.06 (0.88, 1.28)	**1.38 (1.19, 1.59)**	**1.31 (1.06, 1.62)**	**1.51 (1.30, 1.75)**	**1.50 (1.21, 1.85)**
**Health facility**[ii]									
	Health post	0.96 (0.44, 2.08)	2.00 (0.86, 4.66)	1.32 (0.60, 2.87)	1.48 (0.65, 3.38)	**0.26 (0.10, 0.67)**	0.70 (0.25, 2.00)	**0.17 (0.06, 0.45)**	**0.31 (0.11, 0.90)**
	Primary hospital	0.78 (0.49, 1.25)	0.66 (0.39, 1.09)	1.13 (0.71, 1.81)	1.12 (0.69, 1.78)	0.80 (0.50, 1.28)	0.61 (0.36, 1.04)	0.68 (0.42, 1.08)	0.55 (0.33, 0.91)
	General hospital	2.66 (1.51, 4.67)	1.97 (1.08, 3.60)	0.64 (0.37, 1.10)	0.60 (0.34, 1.03)	1.60 (0.93, 2.75)	1.01 (0.54, 1.86)	1.57 (0.91, 2.72)	1.08 (0.59, 1.98)

OR = Odd Ratio
CI = Confidence Interval
AOR = Adjusted Odds Ratio
COR = Core Odds Ratio

[i] Male as reference
[ii] Health center as reference
