# Supplementary material for: Health Care Professionals’ Knowledge, Attitude, Practice, and Infrastructure Accessibility for e-Learning in Ethiopia: Cross-Sectional Study
Source: JMIR Med Educ. 2025 Sep 25;11:e65598. doi: 10.2196/65598 (PMC12463343; doi:10.2196/65598)
Supplement: Multimedia Appendix 7 [file mededu-v11-e65598-s007.pdf]

**KNOWLEDGE**						
		Not confident at all (%)	Slightly confident (%)	Somewhat confident (%)	Fairly confident (%)	Completely confident (%)
	Knowing what e-Learning is.	6.8	13.8	27.4	29.4	22.6
	Ability to do an online search.	9.0	9.5	19.3	26.4	35.7
	Ability to find professional health information on the internet.	7.3	8.8	20.9	28.1	34.9
	Ability to use Microsoft Word (or other text software).	12.8	14.3	25.9	23.4	23.6
	Ability to send emails with attachments.	13.3	14.1	19.1	26.9	26.6
	Ability to create a patient record in the computer.	20.6	11.1	21.4	21.4	25.6
	Ability to analyze data with statistical software.	20.9	21.9	24.9	20.4	12.1
	Ability to participate in online meetings.	25.4	11.8	23.4	18.6	20.9
	Awareness of e-Learning use in the medical education programs.	13.3	11.8	22.6	26.4	25.9
	Awareness of receiving official CPD credits for e-Learning courses.	16.3	13.1	16.8	26.6	27.1
**ATTITUDE**						

		Disagree (%)	Somewhat disagree (%)	Neither agree nor disagree (%)	Agree (%)	Strongly agree (%)
	E-learning is currently possible in rural Ethiopia.	10.3	21.4	12.1	37.4	18.8
	E-learning as a helpful educational tool.	8.5	16.3	10.8	38.7	25.6
	E-Learning should be encouraged in medical teaching institutions in Ethiopia.	0.8	3.5	3.3	31.2	61.3
	E-learning should be used in addition to traditional medical education.	3.3	7.3	8.0	45.2	36.2
	Traditional medical education should be replaced by e-Learning.	4.0	16.8	13.8	39.7	25.6
	I would like to do an e-Learning course rather than a traditional workshop or lecture.	3.8	11.3	7.5	42.5	34.9
	E-Learning is more convenient and flexible than traditional education.	3.3	12.1	8.5	41.5	34.7
	The thought of using e-Learning does not scare me.	10.1	15.6	8.5	47.5	18.3
	E-Learning would be more helpful than problematic for me.	12.1	16.3	14.3	38.2	19.1

	I am willing to put effort into acquiring e-Learning skills, if I don't have them yet.	1.3	2.0	2.5	35.7	58.5	
	I am willing to pay for an e-Learning course in CPD, if I get credits for participating.	6.0	9.3	7.5	40.5	36.7	
	I believe e-Learning would be too expensive for many health professionals.	5.8	7.8	10.1	38.4	37.9	
	I was already waiting for an e-Learning offer in medical education like CPD.	3.8	7.8	11.1	44.7	32.7	
**PRACTICE**							
		Never (%)	Less (%)	Once in the last 3 months (%)	Once a week (%)	Once every few days (%)	At least once a day (%)
	In average I use a smartphone, laptop, desktop or tablet computer …	20.1	13.6	1.3	9.0	15.3	40.7
	In average I use the internet …	16.8	13.3	2.0	7.3	17.3	43.2
	I use the internet to search for general information.	13.3	10.6	1.8	10.3	20.6	43.5
	I use the internet for social networking.	15.8	9.0	1.8	7.5	19.1	46.7
	I use the internet for chatting.	19.3	13.1	3.3	7.5	18.6	38.2
	I use the internet for online money-transactions.	43.2	13.3	4.3	8.3	16.6	14.3

	I use the internet for seeking health and medical-related information.	22.1	12.1	3.5	9.8	23.1	29.4

**ACCESS TO INFRA-STRUCTURE**						
		Never (%)	Almost never (%)	Sometimes (%)	Almost all the time (%)	All the time (%)
	I feel comfortable using English in the medical context.	3.8	10.3	4.3	47.2	34.4
	I have a stable electricity supply.	15.3	6.0	48.5	19.6	10.6
	I can use my private smartphone, laptop, tablet or desktop computer.	23.9	8.5	24.1	13.3	30.2
	I can use a smartphone, laptop, tablet or desktop computer at work.	28.6	10.1	22.6	14.1	24.6
	I have stable internet access outside of my workplace.	36.4	7.3	31.4	10.8	14.1
	I have stable internet access at work.	38.4	8.8	32.7	9.5	10.6
