# Supplementary material for: Health Care Professionals’ Knowledge, Attitude, Practice, and Infrastructure Accessibility for e-Learning in Ethiopia: Cross-Sectional Study
Source: JMIR Med Educ. 2025 Sep 25;11:e65598. doi: 10.2196/65598 (PMC12463343; doi:10.2196/65598)
Supplement: Multimedia Appendix 3 [file mededu-v11-e65598-s003.pdf]

			Health post	Health center	Primary hospital	General hospital	Level	Age in years	Female sex	Nurse	Midwife	Health extension worker	Health officer	General practitioner	Education level	Income group
Spearman-Rho	Health post	Correlation coefficient	1.000	-,236**	-,199**	-,148**	-,148**	0.072	,291**	-,273**	-,132**	,982**	-,124*	-0.096	-,316**	-0.065
		Sig. (2-sided)		0.000	0.000	0.003	0.003	0.152	0.000	0.000	0.008	0.000	0.014	0.056	0.000	0.198
		N	398	398	398	398	398	398	398	398	398	398	398	398	398	398
	Health center	Correlation coefficient	-,236**	1.000	-,555**	-,413**	-,413**	0.079	0.000	0.006	0.027	-,232**	,225**	-,112*	-0.053	-0.077
		Sig. (2-sided)	0.000		0.000	0.000	0.000	0.114	1.000	0.902	0.586	0.000	0.000	0.025	0.294	0.127
		N	398	398	398	398	398	398	398	398	398	398	398	398	398	398
	Primary hospital	Correlation coefficient	-,199**	-,555**	1.000	-,349**	-,349**	-0.083	-,135**	,185**	0.004	-,195**	-,142**	0.028	0.046	-0.018
		Sig. (2-sided)	0.000	0.000		0.000	0.000	0.098	0.007	0.000	0.932	0.000	0.005	0.575	0.363	0.717
		N	398	398	398	398	398	398	398	398	398	398	398	398	398	398
	General hospital	Correlation coefficient	-,148**	-,413**	-,349**	1.000	1,000**	-0.048	-0.037	-0.040	0.049	-,145**	-0.027	,166**	,221**	,156**
		Sig. (2-sided)	0.003	0.000	0.000			0.339	0.458	0.428	0.326	0.004	0.591	0.001	0.000	0.002
		N	398	398	398	398	398	398	398	398	398	398	398	398	398	398
	Level	Correlation coefficient	-,148**	-,413**	-,349**	1,000**	1.000	-0.048	-0.037	-0.040	0.049	-,145**	-0.027	,166**	,221**	,156**
		Sig. (2-sided)	0.003	0.000	0.000			0.339	0.458	0.428	0.326	0.004	0.591	0.001	0.000	0.002
		N	398	398	398	398	398	398	398	398	398	398	398	398	398	398
	Age in years	Correlation coefficient	0.072	0.079	-0.083	-0.048	-0.048	1.000	-,129*	0.055	-,231**	0.063	,184**	-0.080	0.078	,413**
		Sig. (2-sided)	0.152	0.114	0.098	0.339	0.339		0.010	0.273	0.000	0.208	0.000	0.111	0.122	0.000
		N	398	398	398	398	398	398	398	398	398	398	398	398	398	398
	Female sex	Correlation coefficient	,291**	0.000	-,135**	-0.037	-0.037	-,129*	1.000	-0.080	,174**	,286**	-,146**	-,161**	-,378**	-,207**
		Sig. (2-sided)	0.000	1.000	0.007	0.458	0.458	0.010		0.109	0.001	0.000	0.003	0.001	0.000	0.000
		N	398	398	398	398	398	398	398	398	398	398	398	398	398	398

	Nurse	Correlation coefficient	-,273**	0.006	,185**	-0.040	-0.040	0.055	-0.080	1.000	-,456**	-,287**	-,428**	-,331**	-,195**	-,278**
		Sig. (2-sided)	0.000	0.902	0.000	0.428	0.428	0.273	0.109		0.000	0.000	0.000	0.000	0.000	0.000
		N	398	398	398	398	398	398	398	398	398	398	398	398	398	398
	Midwife	Correlation coefficient	-,132**	0.027	0.004	0.049	0.049	-,231**	,174**	-,456**	1.000	-,130**	-,193**	-,150**	-,124*	-,192**
		Sig. (2-sided)	0.008	0.586	0.932	0.326	0.326	0.000	0.001	0.000		0.010	0.000	0.003	0.013	0.000
		N	398	398	398	398	398	398	398	398	398	398	398	398	398	398
	Health extension worker	Correlation coefficient	,982**	-,232**	-,195**	-,145**	-,145**	0.063	,286**	-,287**	-,130**	1.000	-,121*	-0.094	-,327**	-0.066
		Sig. (2-sided)	0.000	0.000	0.000	0.004	0.004	0.208	0.000	0.000	0.010		0.015	0.061	0.000	0.192
		N	398	398	398	398	398	398	398	398	398	398	398	398	398	398
	Health officer	Correlation coefficient	-,124*	,225**	-,142**	-0.027	-0.027	,184**	-,146**	-,428**	-,193**	-,121*	1.000	-,140**	,280**	,307**
		Sig. (2-sided)	0.014	0.000	0.005	0.591	0.591	0.000	0.003	0.000	0.000	0.015		0.005	0.000	0.000
		N	398	398	398	398	398	398	398	398	398	398	398	398	398	398
	General practitioner	Correlation coefficient	-0.096	-,112*	0.028	,166**	,166**	-0.080	-,161**	-,331**	-,150**	-0.094	-,140**	1.000	,435**	,398**
		Sig. (2-sided)	0.056	0.025	0.575	0.001	0.001	0.111	0.001	0.000	0.003	0.061	0.005		0.000	0.000
		N	398	398	398	398	398	398	398	398	398	398	398	398	398	398
	Education level	Correlation coefficient	-,316**	-0.053	0.046	,221**	,221**	0.078	-,378**	-,195**	-,124*	-,327**	,280**	,435**	1.000	,531**
		Sig. (2-sided)	0.000	0.294	0.363	0.000	0.000	0.122	0.000	0.000	0.013	0.000	0.000	0.000		0.000
		N	398	398	398	398	398	398	398	398	398	398	398	398	398	398
	Income group	Correlation coefficient	-0.065	-0.077	-0.018	,156**	,156**	,413**	-,207**	-,278**	-,192**	-0.066	,307**	,398**	,531**	1.000
		Sig. (2-sided)	0.198	0.127	0.717	0.002	0.002	0.000	0.000	0.000	0.000	0.192	0.000	0.000	0.000	
		N	398	398	398	398	398	398	398	398	398	398	398	398	398	398
